# Potential of Fruit Wastes as Natural Resources of Bioactive Compounds

**DOI:** 10.3390/ijms13078308

**Published:** 2012-07-04

**Authors:** Gui-Fang Deng, Chen Shen, Xiang-Rong Xu, Ru-Dan Kuang, Ya-Jun Guo, Li-Shan Zeng, Li-Li Gao, Xi Lin, Jie-Feng Xie, En-Qin Xia, Sha Li, Shan Wu, Feng Chen, Wen-Hua Ling, Hua-Bin Li

**Affiliations:** 1 Guangdong Provincial Key Laboratory of Food, Nutrition and Health, School of Public Health, Sun Yat-Sen University, Guangzhou 510080, China; E-Mails: misyfly@163.com (G.-F.D.); shenchensc1121@163.com (C.S.); kuangrudan@163.com (R.-D.K.); guoyajunleo@163.com (Y.-J.G.); zlslisa@126.com (L.-S.Z.); liligaogll@163.com (L.-L.G.); boscolam24@gmail.com (X.L.); tiger_6219@126.com (J.-F.X.); enqinxia@163.com (E.-Q.X.); lisha0308@hotmail.com (S.L.); wushansw@sina.com (S.W.); lingwh@mail.sysu.edu.cn (W.-H.L.); 2 Key Laboratory of Marine Bio-Resources Sustainable Utilization, South China Sea Institute of Oceanology, Chinese Academy of Sciences, Guangzhou 510301, China; E-Mail: xuxr@scsio.ac.cn; 3 Institute for Food and Bioresource Engineering, College of Engineering, Peking University, Beijing 100871, China; E-Mail: sfchencoe@pku.edu.cn

**Keywords:** fruit residues, FRAP, TEAC, phenolic content

## Abstract

Fruit wastes are one of the main sources of municipal waste. In order to explore the potential of fruit wastes as natural resources of bioactive compounds, the antioxidant potency and total phenolic contents (TPC) of lipophilic and hydrophilic components in wastes (peel and seed) of 50 fruits were systematically evaluated. The results showed that different fruit residues had diverse antioxidant potency and the variation was very large. Furthermore, the main bioactive compounds were identified and quantified, and catechin, cyanidin 3-glucoside, epicatechin, galangin, gallic acid, homogentisic acid, kaempferol, and chlorogenic acid were widely found in these residues. Especially, the values of ferric-reducing antioxidant power (FRAP), trolox equivalent antioxidant capacity (TEAC) and TPC in the residues were higher than in pulps. The results showed that fruit residues could be inexpensive and readily available resources of bioactive compounds for use in the food and pharmaceutical industries.

## 1. Introduction

In the past decade, along with the rise of the middle class and fast economic growth in China, different varieties of fruits produced in China and other countries are increasingly consumed. Due to the high consumption and industrial processing of the edible parts of fruit, fruit wastes such as citrus fruit skins, pineapple residues, sugarcane bagasse and other fruit residues (principally peels and seeds) are generated in large quantities in big cities. Fruit waste has become one of the main sources of municipal solid wastes (MSW), which have been an increasingly tough environmental issue. At present, the two main techniques to dispose MSW are landfill and incineration. However, inappropriate management of landfill will result in emissions of methane and carbon dioxide [1], and incineration involves the subsequent formation and releases of pollutants and secondary wastes such as dioxins, furans, acid gases as well as particulates [2], which pose serious environmental and health risks. For these reasons, there is an urgent need to seek resource and value-added use for fruit wastes. In fact, inexpensive and readily available use of agri-food industry waste is highly cost-effective and minimizes environmental impact. One of the most beneficial approaches is to recover the bioactive constituents, especially the phenolic compounds, making full use of them in the food, pharmaceutical as well as cosmetics industry [3]. Thus, utilization of the fruit wastes as sources of bioactive compounds may be of considerable economic benefits and has become increasingly attractive.

Epidemiological studies indicated that the frequent consumption of fruits is associated with a lower risk of chronic diseases [4–6]. Natural antioxidants in fruits and vegetables, such as vitamins and polyphenols, are considered to be responsible for these health benefits [7,8]. Due to the potential health risks of some synthetic antioxidants [9,10], increasing attention is being paid to identify natural and possibly more economic and effective antioxidants. Phenolic compounds are one of the most important categories of natural antioxidants of interest, and much evidence is derived on the antioxidant potency as well as their prevention of diseases [11–16]. Yet, in recent studies, the antioxidant potency and the content of phenolic compounds were found to be high in the peel and seed of some fruits [17–19], indicating that fruit residues have the potential to be utilized as a resource of bioactive compounds, such as natural antioxidants.

The aim of this study was to systematically evaluate both lipophilic and hydrophilic phenolic contents and their antioxidant potency of wastes (peel and seed) of 50 fruits in order to screen out the residues possessing high antioxidant activities, which could be inexpensive and readily available resources of bioactive compounds for use in food and pharmaceutical industries, ultimately, to find a way out for municipal fruit wastes disposal.

## 2. Results and Discussion

### 2.1. Antioxidant Capacities of Fruit Residues

The FRAP values of the fruit residue extracts are displayed in Table 1. The FRAP values of peel extracts ranged 0.30–68.32, 0.33–104.25, and 0.74–155.73 μmol Fe (II)/g in water-soluble fraction, fat-soluble fraction and total, respectively. The peels with the highest total FRAP values, in decreasing order, were sweetsop peel > mango peel > blueberry peel > Chinese olive peel > hawthorn peel, with total FRAP values of 155.73, 145.43, 104.55, 96.17, and 89.57 μmol Fe(II)/g, respectively. In seed extracts, diverse FRAP values were detected, with ranges of 0.15–96.0, 0.26–85.39, and 0.34–181.39 μmol Fe(II)/g in water-soluble fraction, fat-soluble fraction and total, respectively. Seeds of grape (USA), longan, avocado, ziziphus jujube, and mango presented the highest levels of antioxidant activities, with total FRAP values of 181.39, 86.39, 45.79, 45.53, and 31.17 μmol Fe(II)/g, respectively.

The antioxidant capacities of plant samples could be influenced by many factors, such as extraction solvent and test system, thus it is necessary to perform different evaluation methods to take into account various mechanisms of antioxidant action [20]. In this study, the TEAC assay was used to evaluate free radical scavenging capacities of fruit residues. The TEAC assay is commonly used to determine antioxidant activity of plants and can measure antioxidant capacities of hydrophilic and lipophilic compounds in the same sample [21]. The TEAC values of all peel extracts are given in Table 2. The TEAC values ranged 0.36–46.19, 0.23–50.09, and 1.72–93.10 μmol Trolox/g in water-soluble fraction, fat-soluble fraction and total, respectively. The peels with the highest total TEAC values were ranked as follows: mango peel > sweetsop peel > Chinese olive peel > starfruit peel > hawthorn peel, with TEAC values of 93.10, 84.14, 77.12, 70.15, and 66.76 μmol Trolox/g, respectively. Furthermore, seed extracts were found to present obviously different antioxidant activities. As seen from Table 2, the TEAC values were detected at ranges of 0.19–45.95, 2.23–46.67, and 2.45–92.62 μmol Trolox/g in water-soluble fraction, fat-soluble fraction and total, respectively. Grape seed (USA) extract revealed the highest antioxidant activity, with total TEAC value of 92.62 μmol Trolox/g, followed by longan seed, ziziphus jujuba seed, mango seed, and avocado seed, with total TEAC values of 75.33, 56.03, 50.55, and 42.63 μmol Trolox/g, respectively. In addition, the FRAP and TEAC values of the pulp extracts were also evaluated.

The difference in levels of antioxidant activity between different fruit residues and pulp extracts were statistically analyzed by Friedman and Wilcoxon rank sum test. The results are shown in Table 3. As a whole, the antioxidant capacity of pulp was found to be lower than those of peel and seed extracts. In FRAP assay, a statistically significant difference was detected between fruit residues (peel and seed) and pulp. In TEAC assay, a significant difference was found between peel and pulp. While in both assays, no significant difference was found between peel and seed.

The total antioxidant capacities evaluated by both methods (FRAP and TEAC) revealed similar trends with high correlations. As shown in Table 4, the results exhibited positive linear correlation between them: *R*^2^ in peel and seed were 0.9194 and 0.7821, respectively.

### 2.2. Total Phenolic Content of Fruit Residues

A diverse range of phenolic contents were recorded on the total phenolic contents (TPC) of fruit residues (Table 5). In peel extracts, the phenolic contents were at ranges of 0.10–10.66, 0.06–12.28, and 0.38–22.95 mg GAE/g in water-soluble fraction, fat-soluble fraction and total, respectively. Several peel extracts were found to possess the highest TPC, which, in decreasing order, were mango peel, sweetsop peel, Chinese olive peel, hawthorn peel, and longan pericarp, with the values of 22.95, 17.77, 13.16, 12.66 and 10.92 mg GAE/g, respectively. Moreover, high TPC were also obtained in the seed extracts. Among them, grape seed (USA) was found to be the highest, with a TPC of 22.95 mg GAE/g, followed by longan seed, ziziphus jujuba seed, avocado seed, and mango seed, with phenolic values of 13.58, 9.00, 8.39, and 7.54 mg GAE/g, respectively. The detected ranges of phenolic contents in water-soluble fraction, fat-soluble fraction and total of seed extracts were 0.26–10.82, 0.03–12.14, and 0.30–22.95 mg GAE/g, respectively. Meanwhile, the phenolic contents in pulp extracts were measured as well. Phenolic compounds might tend to accumulate in the dermal tissues of the plant body due to their potential role in protecting against ultraviolet radiations, acting as attractants in fruit dispersal, and as defense chemicals against pathogens and predators [22]. In this study, the total phenolic contents of peel extracts were higher than those of pulp extracts, which was in agreement with the previous study [22]. Additionally, the total phenolic contents in seed extracts were found to be higher than those in pulp extracts, which may be partially because the moisture content of pulps was higher than that in seeds. According to the statistical analysis of phenolic contents among fruit fractions, a statistically significant difference was observed between fruit residues (peel and seed) and pulp (Table 3). The total phenolic contents showed a strong correlation with total antioxidant capacities, indicating that phenolics could be one of the main contributors to the antioxidant capacities of these fruit residues. As summarized in Table 4, the correlation values *R*_2_ between the total FRAP value and TPC value were 0.8775, and 0.9398 for peel and seed, respectively. The correlation values *R*_2_ between the total TEAC value and TPC value were 0.8769 and 0.8556 for peel and seed, respectively. Based on the above discovery, fruit residues—principally peels and seeds—can be a potential source of natural antioxidants. This will not only help bring about commercial benefits, but also help to alleviate environmental pollution problems caused by the poor disposal of such residues.

Among 50 tested fruits, the residues of 12 fruits possessing the strongest antioxidant activities have been screen out based on a combinative consideration of the results of peel and seed obtained from FRAP and TEAC assays as well as the Folin–Ciocalteu method. They were avocado, plantain, blueberry, Chinese olive, grape (USA), guava, hawthorn, longan, mango, starfruit, sweetsop, and ziziphus jujube. Following, the main phenolic compounds and their contents of the residues of these 12 fruit were quantified and the results are shown in Table 6. Catechin, cyanidin 3-glucoside, epicatechin, galangin, gallic acid, homogentisic acid, and kaempferol were widely detected in these residues. The highest contents of catechin were found in grape seed (USA) (241.04 ± 12.54 mg/100 g) and sweetsop peel (143.72 ± 4.22 mg/100 g). Moreover, the highest contents of cyanidin 3-glucoside were found in sweetsop peel (21.00 ± 0.88 mg/100 g). Extremely high levels of epicatechin were recorded in grape seed (USA), hawthorn peel, starfruit peel, and sweetsop peel, with contents of 425.52 ± 19.70, 214.60 ± 8.35, 172.08 ± 6.94, and 164.60 ± 4.30 mg/100 g, respectively. Although gallic acid was detected in most samples, an extraordinarily high level was found in Chinese olive peel (369.60 ± 10.98 mg/100 g), about 34-fold more than the secondly ranked fruit, grape seed (USA) (29.12 ± 1.10 mg/100 g). The contents of kaempferol in 14 samples were >100 mg/100 g, and the maximum value was 160.92 ± 10.55 mg/100 g (ziziphus jujuba peel). Furthermore, extraordinarily high contents of chlorogenic acid (129.44 ± 5.21 mg/100 g) and *p*-hydroxybenzoic acid (68.52 ± 3.88 mg/100 g) in grape seed (USA), forulic acid (52.92 ± 2.85 mg/100 g) in starfruit peel, galangin (109.44 ± 3.98 mg/100 g) in plantain peel, and homogentisic acid (23.40 ± 0.95 mg/100 g) in mango peel were found.

According to the statistical analysis of the antioxidant capacity and total phenolic contents of the fat- and water-soluble fractions from peel and seed extracts by Wilcoxon rank sum test (Table 7), in both peel and seed, TPC, FRAP and TEAC values of fat-soluble fraction were significantly higher than those of water-soluble fraction (*p* < 0.05). Thus, antioxidants in fruit residues are either lipophilic or hydrophilic, and the contribution of lipophilic compounds to antioxidant activity of the fruit residues could not be ignored. When evaluating the total antioxidant potency of fruit residues, both fat-soluble and water-soluble components should be included. However, previous studies only reported the hydrophilic antioxidant components of several fruit residues [23–25], which might underestimate the antioxidant potency of fruit residues.

Although several previous studies have reported the levels of phenolics and antioxidant activity in individual or small groups of fruit residues [26–28], the diversity of extraction and analysis methods makes it difficult to directly compare the results from different investigations. To our knowledge, our study systematically evaluated both lipophilic and hydrophilic phenolic contents and their antioxidant potency of residues of 50 fruits, and was the largest scale such study, providing comprehensive information for the future value-added utilization of fruit residues.

## 3. Experimental Section

### 3.1. Chemicals and Samples

6-Hydroxy-2,5,7,8-tetramethylchromane-2-carboxylic acid (Trolox), 2,4,6-Tri(2-pyridyl)-*S*-triazine (TPTZ), 2,2′-azinobis(3-ethylbenothiazoline-6-sulfonic acid) diammonium salt (ABTS) and Folin–Ciocalteu’s phenol reagent were purchased from Sigma–Aldrich (St. Louis, MO, USA). The standard compounds gallic acid, catechin, chlorogenic acid, cyanidin 3-glucoside, epicatechin, forulic acid, galangin, homogentisic acid, kaempferol, p-hydroxybenzoic acid, protocatechuic acid, and tangeretin were bought from Sigma–Aldrich and Siyi Biotechnology Company (Chengdu, China). Methanol was HPLC grade and purchased from Merck (Germany). Acetic acid, hydrochloric acid, tetrahydrofuran, potassium persulfate, iron (III) chloride 6-hydrate, iron (II) sulfate 7-hydrate, sodium acetate and sodium carbonate were analytical grade and obtained from Tianjin Chemical Factory (Tianjin, China). Deionized water was used throughout the experiment.

The stock solutions of the standard compounds (10 mg/mL) were prepared in methanol, and stored at 4 °C. The calibration standards (5–100 μg/mL) were prepared from the stock solution by the serial dilution of methanol.

Fruit samples were collected from markets in Guangzhou, China.

### 3.2. Sample Preparation

The fresh fruits were cleaned with deionized water and then separated into peel, seed and pulp. Immediately, the separated fruit fractions were ground into fine particles with a special grinder. Hydrophilic and lipophilic components of fruit fractions were extracted as previously reported, with minor modifications [29]. Briefly, 0.5 g precisely weighed sample was extracted with 5 mL of tetrahydrofuran in a shaking water bath (100 rpm, 37 °C) for 30 min. Then the mixture was centrifuged at 4200 g for 30 min, and the supernatant was recovered. The extraction was repeated once with 5 mL of tetrahydrofuran under the same condition and the two supernatants were combined into fat-soluble fraction. Subsequently, the residue was extracted twice with methanol-acetic acid-water mixture (50:3.7:46.3, *v*/*v*) (5 mL each time) in a shaking water bath (100 rpm, 37 °C) for 30 min and the two supernatants were combined into water-soluble fraction. The extracts were stored at −20 °C and measured within 24 h.

### 3.3. Ferric-Reducing Antioxidant Power (FRAP) Assay

The FRAP assay was performed based on the procedure described by Benzie and Strain [30] with slight modifications. In this assay, 100 μL of the diluted sample was added to 3 mL of the FRAP reagent and the reaction was monitored after 4 min at 593 nm. The results were expressed as micromole Fe (II)/g wet weight of fruit residue.

### 3.4. Trolox Equivalent Antioxidant Capacity (TEAC) Assay

The TEAC assay was performed according to the method established previously [21] with minor modifications. Briefly, the ABTS•+ stock solution was prepared from 7 mmol/L ABTS and 2.45 mmol/L potassium persulfate in a volume ratio of 1:1, and then incubated in the dark at room temperature for 16 h and used within 2 days. A 100 μL of the tested sample was mixed with 3.8 mL ABTS•+ working solution and the absorbance was taken at 734 nm after 6 min of incubation at room temperature. The percent of inhibition of absorbance at 734 nm was calculated and the results were expressed as micromole Trolox/g wet weight of fruit residue.

### 3.5. Determination of Total Phenolic Content

Total phenolic contents were determined with Folin–Ciocalteu method [31]. Briefly, 0.50 mL extract was mixed with 2.5 mL of 1:10 diluted Folin–Ciocalteu reagent. After 4 min, 2 mL of saturated sodium carbonate solution was added. The mixture was incubated in dark for 2 h at room temperature and its absorbance was detected at 760 nm. Gallic acid was used for calibration, and the results were expressed as mg of gallic acid equivalent (mg GAE) per 100 g wet weight of fruit residue.

### 3.6. HPLC Analysis

The phenolic ingredients in fruit residue samples were analyzed by HPLC-PAD according to the method illustrated by Sakakibara and his colleagues [32] with small modification. In brief, the HPLC system employed a Waters (Milford, MA, USA) 1525 binary HPLC pump separation module equipped with an auto-injector and a Waters 2996 photodiode array detector. Separation was performed with an Agilent Zorbax Extend-C18 column (250 mm × 4.6 mm, 5 μm) at 35 °C with a gradient elution solution A, comprising acetic acid-water solution (0.1% acetic acid) and methanol (9:1; *v*/*v*), and solution B, composed of methanol and acetic acid-water solution (0.1% acetic acid) (7:3; *v*/*v*), which delivered at a flow rate of 1.0 mL/min as follows: 0 min, 100% (A); 15 min, 70% (A); 45 min, 65% (A); 65 min, 60% (A); 70 min, 50% (A); and 95 min, 0% (A). The UV spectra were recorded between 190 and 600 nm for peak characterization. Phenolic ingredients were quantified by the peak area of maximum absorption wavelength.

### 3.7. Statistical Analysis

All the experiments were performed in triplicate, and the results were expressed as mean ± SD (standard deviation). Statistical analysis was performed using SPSS 13.0 and Excel 2003. In order to investigate the difference in levels of antioxidant activity and phenolics between different fruit residues, statistical analyses were carried out using Friedman and Wilcoxon rank sum test.

## 4. Conclusions

The antioxidant capacities, phenolic contents and their correlation, for water- and fat-soluble extracts of the residues of 50 fruits were studied in detail. Fruits residues possessing strongest antioxidant properties were screen out. Positive correlation between antioxidant potency and total phenolic content indicated that phenolics could be one of the main contributors to the antioxidant capacities of these fruit residues. The values of FRAP, TEAC and TPC in peels and seeds were higher than those in pulps, indicating that they could be inexpensive and readily available resources of bioactive compounds (such as natural antioxidant) for use in the food and pharmaceutical industries.

## Figures and Tables

**Table 1:** Ferric-Reducing Antioxidant Power (FRAP) values of fruit residues.

Fruits	FRAP values (μmol Fe(II)/g) of Peel			FRAP values (μmol Fe(II)/g) of Seed		
	
	Fat-soluble fraction	Water-soluble fraction	Total	Fat-soluble fraction	Water-soluble fraction	Total
Apple (green)	4.26 ± 0.16	6.20 ± 0.25	10.46 ± 0.42	3.21 ± 0.11	2.33 ± 0.18	5.54 ± 0.29
Apple (red)	4.45 ± 0.43	6.88 ± 0.71	11.33 ± 1.14	4.05 ± 0.57	3.90 ± 0.21	7.95 ± 0.78
Avocado	25.34 ± 2.27	13.16 ± 2.53	38.49 ± 4.80	28.99 ± 2.68	16.80 ± 0.33	45.79 ± 3.01
Banana	2.36 ± 0.10	1.21 ± 0.33	3.57 ± 0.43	-	-	-
Black plum	25.59 ± 0.87	6.59 ± 0.81	32.17 ± 1.68	10.44 ± 1.52	1.48 ± 0.35	11.92 ± 1.87
Blueberry	62.56 ± 0.18	41.99 ± 0.59	104.55 ± 0.77	-	-	-
Cherry	8.52 ± 0.20	7.83 ± 0.50	16.35 ± 0.70	0.41 ± 0.05	nd	0.41 ± 0.05
Cherry tomatoes	4.30 ± 0.05	0.76 ± 0.01	5.06 ± 0.06	-	-	-
Chinese olive	62.93 ± 1.95	33.24 ± 4.73	96.17 ± 6.69	-	-	-
Durian	-	-	-	2.90 ± 0.53	4.95 ± 0.21	7.85 ± 0.74
Garland fruit	7.41 ± 1.21	6.73 ± 0.25	14.14 ± 1.46	3.12 ± 0.10	nd	3.12 ± 0.10
Ginseng fruit	4.29 ± 0.33	3.11 ± 0.15	7.39 ± 0.48	-	-	-
Grape (green)	2.52 ± 0.30	23.06 ± 1.77	25.58 ± 2.07	-	-	-
Grape (USA)	21.26 ± 2.19	13.71 ± 0.51	34.98 ± 2.70	85.39 ± 2.07	96.00 ± 4.86	181.39 ± 6.93
Grapefruit	11.76 ± 0.37	6.04 ± 0.45	17.80 ± 0.82	-	-	-
Greengage	10.54 ± 1.11	7.36 ± 1.46	17.91 ± 2.58	3.74 ± 0.30	1.66 ± 0.01	5.40 ± 0.31
Guava	42.36 ± 5.56	11.76 ± 0.44	54.12 ± 5.99	9.18 ±0.66	5.46 ± 0.56	14.64 ± 1.21
Hawthorn	55.33 ± 3.51	39.24 ± 3.03	89.57 ± 6.54	6.02 ± 0.30	5.16 ± 0.36	11.19 ± 0.66
Jackfruit	-	-	-	6.21 ± 0.30	2.21 ± 0.20	8.43 ± 0.51
Kiwi fruit	9.24 ± 0.10	6.66 ± 0.51	15.90 ± 0.61	-	-	-
Lemon	1.79 ± 0.20	3.97 ± 0.45	5.76 ± 0.66	0.86 ± 0.10	0.51 ± 0.01	1.37 ± 0.11
Longan	36.73 ± 3.49	26.55 ± 1.82	63.28 ± 5.30	56.60 ± 0.73	29.79 ± 1.30	86.39 ± 2.03
Loquat	1.40 ± 0.27	1.46 ± 0.25	2.87 ± 0.52	7.62 ± 0.18	4.95 ± 0.41	12.57 ± 0.59
Mandarin orange	3.64 ± 0.06	6.35 ± 0.59	9.99 ± 0.65	7.85 ± 0.85	4.96 ± 0.10	12.81 ± 0.94
Mango	77.11 ± 14.70	68.32 ± 3.89	145.43 ± 18.59	24.29 ± 1.80	6.88 ± 1.82	31.17 ± 3.62
Mangosteen	18.40 ± 0.15	9.46 ± 0.55	27.86 ± 0.71	5.10 ± 0.37	6.26 ± 0.05	11.36 ± 0.42
Muskmelon (yellow)	6.26 ± 0.40	6.79 ± 0.45	13.05 ± 0.86	0.61 ± 0.01	nd	0.61 ± 0.01
Navel orange (USA)	16.07 ± 1.82	7.73 ± 0.05	23.80 ±1.87	-	-	-
Nectarine (China)	1.23 ± 0.15	nd	1.23 ± 0.15	0.34 ± 0.01	nd	0.34 ± 0.01
Nectarine (USA)	11.93 ± 0.59	9.60 ± 1.30	21.53 ± 1.89	19.43 ± 0.24	8.39 ± 0.76	27.82 ± 1.00
Netted melon	3.79 ± 0.15	3.29 ± 0.15	7.09 ± 0.30	nd	nd	nd
Pawpaw	2.19 ± 0.30	1.71 ± 0.04	3.90 ± 0.34	4.97 ± 0.20	2.54 ± 0.20	7.51 ± 0.40
Peach (honey)	3.73 ± 0.29	3.66 ± 0.34	7.38 ± 0.63	3.85 ± 0.47	7.23 ± 0.88	11.08 ± 1.36
Pear (fragrant)	8.10 ± 0.24	7.10 ± 0.24	15.20 ± 0.47	4.35 ± 0.24	4.52 ± 0.35	8.87 ± 0.59
Pear (crystal)	4.95 ± 0.19	4.66 ± 0.43	9.61 ± 0.62	16.27 ± 0.56	6.90 ± 0.72	23.17 ± 1.27
Peer (red, USA)	7.84 ± 1.06	6.51 ± 0.40	14.35 ± 1.46	9.91 ± 1.26	3.69 ± 0.15	13.60 ± 1.41
Pineapple	3.59 ± 0.40	2.24 ± 0.25	5.83 ± 0.65	-	-	-
Pitaya	0.33 ± 0.05	0.76 ± 0.05	1.09 ± 0.10	-	-	-
Plantain	5.23 ± 0.18	6.93 ± 0.12	12.16 ± 0.29	-	-	-
Plum (red, Australia)	12.55 ± 1.16	3.87 ± 0.81	16.42 ± 1.97	8.48 ± 0.15	1.87 ± 0.26	10.35 ± 0.41
Pomelo (golden)	4.24 ± 0.14	0.85 ± 0.08	5.09 ± 0.23	-	-	-
Pomelo (green)	9.81 ± 0.43	7.02 ± 0.71	16.83 ± 1.14	3.59 ± 0.10	1.81 ± 0.20	5.40 ± 0.30
Plumcot	-	-	-	12.50 ± 0.91	1.98 ± 0.08	14.48 ± 0.99
Snake fruit	0.44 ± 0.05	0.30 ± 0.05	0.74 ± 0.10	-	-	-
Starfruit	31.52 ± 0.46	53.00 ± 2.73	84.52 ± 3.19	15.04 ± 2.68	2.36 ± 0.20	17.39 ± 2.88
Sweetsop	104.25 ± 5.68	51.48 ± 2.78	155.73 ± 8.46	12.09 ± 1.52	3.55 ± 0.45	15.64 ± 1.97
Tangerine	13.31 ± 0.06	1.68 ± 0.33	14.99 ± 0.39	7.31 ± 0.41	4.18 ± 0.36	11.49 ± 0.78
Watermelon	0.54 ± 0.05	0.90 ± 0.15	1.44 ± 0.20	0.26 ± 0.05	0.15 ± 0.01	0.41 ± 0.06
Wax-apple	2.01 ± 0.30	6.72 ± 0.50	8.73 ± 0.80	-	-	-
Ziziphus jujuba	2.52 ± 0.11	5.94 ± 0.96	8.45 ± 1.07	22.19 ± 0.85	23.34 ± 0.35	45.53 ± 1.21

nd: not detected.

**Table 2:** Trolox Equivalent Antioxidant Capacity (TEAC) values of fruit residues.

Fruits	TEAC values (μmol Trolox/g) of Peel			TEAC values (μmol Trolox/g) of seed		
	
	Fat-soluble fraction	Water-soluble fraction	Total	Fat-soluble fraction	Water-soluble fraction	Total
Apple (green)	9.42 ± 0.25	5.45 ± 0.44	14.87 ± 0.68	11.33 ± 0.61	1.62 ± 0.20	12.96 ± 0.82
Apple (red)	10.72 ± 1.11	4.77 ± 0.8	15.49 ± 1.91	13.55 ± 0.68	2.59 ± 0.18	16.14 ± 0.86
Avocado	23.18 ± 2.30	11.55 ± 1.12	34.72 ± 3.42	30.20 ± 1.28	12.43 ± 0.66	42.63 ± 1.94
Banana	nd	nd	nd	-	-	-
Black plum	20.06 ± 0.13	6.32 ± 0.57	26.38 ± 0.70	15.97 ± 1.84	2.01 ± 0.13	3.02 ± 1.97
Blueberry	39.69 ± 2.04	19.41 ± 1.82	59.10 ± 3.85	12.80 ± 0.67	1.15 ± 0.06	13.96 ± 0.73
Cherry	17.62 ± 1.12	6.53 ± 0.80	24.15 ± 1.91	13.68 ± 1.12	0.51 ± 0.01	14.19 ± 1.13
Cherry tomatoes	4.93 ± 0.17	1.42 ± 0.36	6.35 ± 0.53	-	-	-
Chinese olive	46.52 ± 0.32	30.60 ± 2.11	77.12 ± 2.43	-	-	-
Durian	-	-	-	27.35 ± 0.91	4.90 ± 0.16	32.25 ± 1.07
Garland fruit	10.23 ± 1.08	5.49 ± 0.40	15.72 ± 1.48	9.15 ± 0.45	0.44 ± 0.17	9.59 ± 0.62
Ginseng fruit	3.47 ± 0.01	nd	3.47 ± 0.01	-	-	-
Grape (green)	9.95 ± 0.05	24.56 ± 3.35	34.51 ± 3.39	-	-	-
Grape (USA)	24.69 ± 0.40	8.6 ± 0.23	33.30 ± 0.62	46.67 ± 0.17	45.95 ± 0.20	92.62 ± 0.37
Grapefruit	20.71 ± 1.91	nd	20.71 ± 1.91	-	-	-
Greengage	10.93 ± 0.64	2.30 ± 0.21	13.23 ± 0.85	7.42 ± 0.78	nd	7.42 ± 0.78
Guava	30.49 ± 3.56	5.87 ± 0.11	36.36 ± 3.68	6.28 ± 0.42	5.87 ± 0.01	12.15 ± 0.43
Hawthorn	40.86 ± 2.55	25.90 ± 0.22	66.76 ± 2.77	8.37 ± 1.01	2.56 ± 0.22	10.93 ± 1.24
Jackfruit	-	-	-	4.19 ± 0.45	nd	4.19 ± 0.45
Kiwi fruit	15.04 ± 1.16	5.59 ± 0.12	20.63 ± 1.28	-	-	-
Lemon	2.10 ± 0.12	2.97 ± 0.12	5.06 ± 0.25	-	-	-
Longan	37.68 ± 2.44	24.52 ± 3.01	62.19 ± 5.45	46.01 ± 0.19	29.32 ± 2.58	75.33 ± 2.76
Loquat	3.75 ± 0.62	nd	3.75 ± 0.62	nd	nd	nd
Mandarin orange	5.65 ± 0.28	2.96 ± 0.11	8.61 ± 0.40	3.00 ±0.17	1.35 ± 0.11	4.35 ± 0.28
Mango	46.91 ± 0.44	46.19 ± 0.48	93.10 ± 0.93	42.39 ± 1.33	8.16 ± 1.19	50.55 ± 2.52
Mangosteen	19.01 ± 0.92	10.30 ± 0.48	29.31 ± 1.40	6.04 ± 0.48	6.79 ± 1.48	12.83 ± 1.95
Muskmelon (yellow)	6.21 ± 0.21	0.80 ± 0.07	7.01 ± 0.28	2.45 ± 0.57	nd	2.45 ± 0.57
Navel orange (USA)	9.60 ± 0.82	2.49 ± 0.23	12.09 ± 1.05	-	-	-
Nectarine (China)	6.42 ± 0.68	0.36 ± 0.06	6.78 ± 0.74	2.65 ± 0.34	nd	2.65 ± 0.34
Nectarine (USA)	15.80 ± 1.14	4.69 ± 0.17	20.49 ± 1.31	14.80 ± 0.62	4.16 ± 0.23	18.94 ± 0.85
Netted melon	4.21 ± 0.64	nd	4.21 ± 0.64	6.86 ± 1.14	nd	6.86 ± 1.14
Pawpaw	4.76 ± 0.43	nd	4.76 ± 0.43	9.77 ± 0.14	nd	9.77 ± 0.14
Peach (honey)	10.95 ± 0.62	nd	10.95 ± 0.62	14.32 ± 1.65	5.33 ± 0.40	19.64 ± 2.04
Pear (fragrant)	4.48 ± 0.45	nd	4.48 ± 0.45	8.66 ± 0.57	2.12 ± 0.06	10.78 ± 0.62
Pear (crystal)	10.29 ± 0.61	3.68 ± 0.12	13.97 ± 0.74	18.29 ± 0.57	7.33 ± 0.86	25.62 ± 1.43
Peer (red, USA)	10.89 ± 1.65	5.54 ± 0.26	16.44 ± 1.91	14.38 ± 0.66	3.73 ± 0.33	18.11 ± 0.99
Pineapple	4.45 ± 0.45	2.56 ± 0.21	7.01 ± 0.65	-	-	-
Pitaya	0.23 ± 0.06	1.49 ± 0.25	1.72 ± 0.31	-	-	-
Plantain	12.62 ± 0.94	0.71 ± 0.11	13.33 ± 1.05	-	-	-
Plum (red, Australia)	13.87 ± 0.59	3.15 ± 0.51	17.03 ± 1.10	12.80 ± 0.25	1.77 ± 0.20	14.58 ± 0.44
Pomelo (golden)	10.88 ± 0.50	1.02 ± 0.06	11.90 ± 0.56	-	-	-
Pomelo (green)	17.03 ± 0.43	4.96 ± 0.10	21.98 ± 0.53	13.46 ± 0.31	2.94 ± 0.31	16.40 ± 0.61
Plumcot	11.41 ± 0.45	1.34 ± 0.28	12.75 ± 0.74	10.49 ± 0.85	nd	10.49 ± 0.85
Snake fruit	3.57 ± 0.17	0.93 ± 0.05	4.50 ± 0.22	-	-	-
Starfruit	34.66 ± 1.02	35.49 ± 3.89	70.15 ± 4.91	4.95 ± 0.51	6.20 ± 0.01	11.15 ± 0.52
Sweetsop	54.09 ± 0.14	30.05 ± 2.77	84.14 ± 2.91	8.37 ± 1.28	0.38 ± 0.07	8.74 ± 1.09
Tangerine	14.28 ± 1.37	nd	14.28 ± 1.37	7.48 ± 1.11	0.19 ± 0.06	7.67 ± 1.16
Watermelon	0.92 ± 0.06	1.01 ± 0.18	1.93 ± 0.25	2.23 ± 0.06	0.79 ± 0.12	3.02 ± 0.18
Wax-apple	0.24 ± 0.05	6.47 ± 0.22	6.72 ± 0.27	-	-	-
Ziziphus jujuba	12.98 ± 1.70	1.10 ± 0.21	14.08 ± 1.92	30.41 ± 1.04	25.62 ± 0.99	56.03 ± 2.04

nd: not detected

**Table 3:** Comparison of antioxidant activity levels and phenolic contents of peel, seed and pulp.

Item	Fraction	No.	Mean ± SD	Median (25th, 75th)	*Z*	*p*
ABTS	peel	30	23.78 ± 24.08	15.18 (7.01, 30.31)	−4.271 a	<0.001 a
	seed	30	17.49 ± 19.45	11.65 (7.28, 18.32)	−1.553 b	0.120 b
	pulp	30	8.50 ± 7.51	7.84 (3.64, 11.30)	−2.335 c	0.020 c
FRAP	peel	30	29.55 ± 39.32	8.18 (14.67, 32.87)	−4.762 a	<0.001 a
	seed	30	18.58 ± 32.88	11.28 (5.51, 16.08)	−1.841 b	0.066 b
	pulp	30	5.83 ± 8.43	3.55 (0.86, 7.57)	−3.492 c	<0.001 c
TPC	peel	30	5.77 ± 4.73	4.15 (3.61, 6.83)	−4.782 a	<0.001 a
	seed	30	4.55 ± 3.95	3.65 (2.75, 4.81)	−1.718 b	0.086 b
	pulp	30	2.36 ± 1.38	2.45 (1.78, 2.98)	−3.980 c	<0.001 c

A calculated P value was considered to be statistically significant difference at level of 1.7% (Inspection level was adjusted to α = 0.017).

a comparison of peel and pulp;

b comparison of peel and seed;

c comparison of seed and pulp.

**Table 4:** The relationships between several evaluation parameters.

Portion	Parameter	Water-soluble fraction	Fat-soluble fraction	Total
Peel	FRAP value *vs.* TEAC value	*Y* = 1.36*X* + 1.25 *R*_2_ = 0.9173	*Y* = 1.*53X* − 7.20 *R*_2_ = 0.8737	*Y* = 1.49*X* − 6.36 *R*_2_ = 0.9194
	FRAP value *vs.* Total phenolic content	*Y* = 7.*29X* − 0.93 *R*_2_ = 0.9052	*Y* = 7.90*X* − 12.81 *R*_2_ = 0.8067	*Y* = 7.90*X* − 14.76 *R*_2_ = 0.8775
	TEAC value *vs.* Total phenolic content	Y = 5.06*X* − 1.17 *R*_2_ = 0.8703	*Y* = 4.87*X* − 2.53 *R*_2_ = 0.8153	*Y* = 5.10*X* − 4.60 *R*_2_ = 0.8769
Seed	FRAP value *vs.* TEAC value	*Y* = 1.60*X* − 0.83 *R*_2_ = 0.855	Y = 1.11*X* − 3.27 *R*_2_ = 0.6443	*Y* = 1.40*X* − 7.00 *R*_2_ = 0.7821
	FRAP value *vs.* Total phenolic content	*Y* = 8.61*X* − 3.08 *R*_2_ = 0.9706	*Y* = 6.98*X* − 12.16 *R*_2_ = 0.8604	*Y* = 7.96*X* − 17.81 *R*_2_ = 0.9398
	TEAC value *vs.* Total phenolic content	*Y* = 4.*80X* − 0.69 *R*_2_ = 0.8933	Y = 4.70*X* − 2.37 *R*_2_ = 0.7497	*Y* = 4.*81X* − 3.45 *R*_2_ = 0.8556

**Table 5:** Total phenolic contents of fruit residues.

Fruits	TPC (mg GAE/g) of Peel			TPC (mg GAE/g) of seed		
	
	Fat-soluble fraction	Water-soluble fraction	Total	Fat-soluble fraction	Water-soluble fraction	Total
Apple (green)	2.82 ± 0.09	1.07 ± 0.02	3.89 ± 0.12	3.13 ± 0.41	0.51 ± 0.06	3.64 ± 0.47
Apple (red)	3.04 ± 0.21	1.33 ± 0.15	4.37 ± 0.37	3.43 ± 0.15	1.08 ± 0.08	4.51 ± 0.23
Avocado	5.12 ± 0.61	2.08 ± 0.02	7.20 ± 0.64	5.73 ± 0.19	2.66 ± 0.08	8.39 ± 0.27
Banana	0.89 ± 0.18	0.14 ± 0.01	1.02 ± 0.19	-	-	-
Black plum	3.12 ± 0.16	1.04 ± 0.14	4.16 ±0.30	3.85 ± 0.30	0.43 ± 0.05	4.28 ± 0.34
Blueberry	5.79 ± 0.14	3.13 ± 0.10	8.92 ± 0.24	-	-	-
Cherry	3.11 ± 0.24	1.21 ± 0.10	4.32 ±0.34	2.32 ± 0.10	0.40 ± 0.12	2.72 ± 0.21
Cherry tomatoes	2.72 ± 0.13	0.35 ± 0.08	3.07 ± 0.21	-	-	-
Chinese olive	8.76 ±0.06	4.40 ± 0.25	13.16 ± 0.32	-	-	-
Durian	-	-	-	2.70 ± 0.18	0.96 ±0.08	3.67 ± 0.26
Garland fruit	2.91 ± 0.18	1.21 ± 0.03	4.13 ± 0.21	3.12 ± 0.06	0.85 ±0.09	3.97 ± 0.15
Ginseng fruit	0.86 ± 0.08	0.35 ± 0.03	1.21 ± 0.11	-	-	-
Grape (green)	2.87 ± 0.004	1.91 ± 0.30	4.78 ± 0.31	-	-	-
Grape (USA)	5.85 ± 0.27	2.30 ± 0.14	8.15 ± 0.41	12.14 ± 0.13	10.82 ±0.88	22.95 ± 1.01
Grapefruit	4.89 ± 0.18	2.00 ± 0.17	6.89 ± 0.35	-	-	-
Greengage	4.05 ±0.08	1.48 ± 0.10	5.53 ± 0.18	2.87 ± 0.32	0.83 ±0.08	3.70 ± 0.39
Guava	5.55 ± 0.66	1.71 ± 0.21	7.26 ± 0.87	1.34 ±0.17	0.34 ± 0.07	1.68 ± 0.24
Hawthorn	8.19 ± 0.72	4.47 ± 0.37	12.66 ±1.09	2.83 ± 0.04	0.83 ± 0.03	3.66 ± 0.07
Jackfruit	-	-	-	1.38 ± 0.25	1.06 ± 0.02	2.43 ± 0.27
Kiwi fruit	3.36 ± 0.03	0.90 ± 0.04	4.26 ± 0.07	-	-	-
Lemon	0.59 ± 0.10	1.40 ± 0.08	1.99 ± 0.18	8.95 ±0.08	4.63 ± 0.35	13.58 ±0.43
Longan	6.47 ± 0.01	4.45 ± 0.31	10.92 ± 0.32	1.52 ± 0.28	0.85 ±0.05	2.37 ± 0.32
Loquat	0.45 ± 0.09	0.24 ± 0.005	0.68 ± 0.10	-	-	-
Mandarin orange	2.86 ±0.37	0.78 ± 0.07	3.64 ±0.44	2.17 ± 0.25	0.61 ±0.02	2.77 ± 0.26
Mango	12.28 ± 0.32	10.66 ± 0.78	22.95 ± 1.09	6.05 ± 0.21	1.49 ± 0.02	7.54 ± 0.24
Mangosteen	4.31 ± 0.07	2.40 ± 0.05	6.71 ± 0.12	0.95 ± 0.08	1.42 ± 0.12	2.38 ± 0.21
mulberry	-	-	-	-	-	-
Muskmelon (yellow)	3.98 ± 0.48	1.12 ± 0.13	5.10 ± 0.61	-	-	-
Navel orange (USA)	4.15 ± 0.06	1.56 ±0.13	5.71 ± 0.19	2.69 ± 0.25	0.34 ± 0.03	3.03 ± 0.28
Nectarine (China)	2.17 ±0.06	0.10 ±0.01	2.28 ± 0.07	1.95 ± 0.13	nd	1.95 ±0.13
Nectarine (USA)	3.47 ± 0.29	1.24 ± 0.09	4.71 ± 0.38	4.03 ± 0.61	1.01 ± 0.12	5.04 ± 0.72
Netted melon	3.00 ± 0.11	0.65 ±0.07	3.65 ±0.18	2.44 ± 0.06	0.32 ± 0.01	2.76 ± 0.08
Pawpaw	2.78 ± 0.13	0.71 ± 0.09	3.49 ± 0.23	2.69 ± 0.11	0.68 ± 0.05	3.37 ± 0.16
Peach (honey)	2.56 ±0.03	0.21 ± 0.02	2.77 ± 0.05	2.59 ± 0.29	1.75 ±0.01	4.34 ± 0.30
Pear (fragrant)	2.80 ±0.06	0.86 ± 0.05	3.67 ± 0.11	2.82 ± 0.19	0.55 ± 0.02	3.36 ± 0.21
Pear (crystal)	2.95 ± 0.16	0.63 ± 0.03	3.57 ± 0.19	4.23 ± 0.13	0.95 ± 0.05	5.17 ± 0.18
Peer (red, USA)	2.96 ± 0.14	1.18 ± 0.10	4.14 ±0.24	3.90 ± 0.19	0.83 ± 0.06	4.73 ± 0.25
Pineapple	2.66 ± 0.15	0.96 ±0.03	3.62 ± 0.18	-	-	-
Pitaya	0.06 ± 0.01	0.32 ± 0.05	0.38 ± 0.05	-	-	-
Plantain	3.25 ± 0.08	0.40 ± 0.05	3.65 ± 0.14	-	-	-
Plum (red, Australia)	3.28 ± 0.21	0.67 ± 0.09	3.95 ± 0.30	3.15 ± 0.22	0.46 ±0.06	3.61 ± 0.29
Pomelo (golden)	3.56 ± 0.03	1.23 ± 0.01	4.79 ± 0.05	-	-	-
Pomelo (green)	3.34 ± 0.14	0.92 ± 0.07	4.25 ± 0.21	2.90 ± 0.03	0.26 ± 0.01	3.16 ± 0.05
Plumcot	2.89 ± 0.33	1.38 ± 0.15	4.26 ± 0.48	3.09 ± 0.38	0.33 ±0.03	3.42 ± 0.42
Snake fruit	2.59 ±0.36	0.69 ± 0.06	3.28 ±0.42	-	-	-
Starfruit	3.31 ± 0.33	7.14 ± 0.64	10.45 ±0.97	3.53 ± 1.16	0.55 ± 0.15	4.08 ±1.32
Sweetsop	11.63 ± 0.04	6.14 ± 0.12	17.77 ± 0.16	4.92 ± 0.12	0.89 ±0.10	5.81 ± 0.22
Tangerine	3.28 ± 0.07	0.24 ± 0.004	3.52 ± 0.08	2.38 ± 0.16	0.64 ± 0.05	3.02 ± 0.22
Watermelon	0.21 ± 0.003	0.37 ± 0.01	0.58 ± 0.01	0.03 ± 0.01	0.26 ±0.02	0.30 ± 0.03
Wax-apple	0.12 ± 0.01	0.84 ± 0.01	0.96 ± 0.02	-	-	-
Ziziphus jujuba	2.82 ± 0.16	1.48 ± 0.22	4.30 ± 0.38	5.60 ± 0.75	3.40 ± 0.19	9.00 ± 0.94

nd: not detected.

**Table 6:** Main phenolic compounds and their contents (mean ± SD, mg/100 g) of residues from 12 fruits with highest antioxidant activities.

Fruits	Peel		Seed	
	
	Phenolics	Contents	Phenolics	Contents
Avocado	Epicatechin	21.92 ± 0.78	Catechin	52.08 ± 1.66
	Gallic acid	5.20 ± 0.20	Chlorogenic acid	11.68 ± 0.74
	-	-	Cyanidin 3-glucoside	3.16 ± 0.12
	-	-	Homogentisic acid	11.36 ± 0.54
Plantain	Galangin	109.44 ± 3.98	-	-
	Gallic acid	10.08 ± 0.52	-	-
Blueberry	Catechin	61.16 ± 3.63	-	-
	Chlorogenic acid	4.48 ± 0.20	-	-
	Cyanidin 3-glucoside	5.72 ± 0.30	-	-
	Epicatechin	54.80 ± 3.25	-	-
	Gallic acid	14.24 ± 0.82	-	-
Chinese olive	Cyanidin 3-glucoside	5.88 ± 0.24	-	-
	Epicatechin	27.68 ± 1.32	-	-
	Gallic acid	369.60 ± 10.98	-	-
	Homogentisic acid	12.64 ± 0.77	-	-
	Tangeretin	266.52 ± 9.90	-	-
Grape (USA)	Catechin	25.96 ± 0.67	Catechin	241.04 ± 12.54
	Gallic acid	10.00 ± 0.25	Chlorogenic acid	129.44 ± 5.21
	Kaempferol	154.72 ± 8.10	Cyanidin 3-glucoside	11.08 ± 0.41
	-	-	Epicatechin	425.52 ± 19.70
	-	-	Gallic acid	29.12 ± 1.10
	-	-	Homogentisic acid	15.28 ± 0.65
	-	-	P-hydroxybenzoic acid	68.52 ± 3.88
	-	-	Protocatechuic acid	14.76 ± 0.40
Guava	Catechin	31.48 ± 1.55	Cyanidin 3-glucoside	3.12 ± 0.10
	Galangin	74.68 ± 4.42	Gallic acid	5.56 ± 0.20
	Homogentisic acid	9.88 ± 0.70	Kaempferol	154.64 ± 7.85
Hawthorn	Catechin	57.80 ± 4.30	Kaempferol	154.56 ± 6.32
	Cyanidin 3-glucoside	5.92 ± 0.22	Gallic acid	10.04 ± 0.40
	Epicatechin	214.60 ± 8.35	-	-
	Kaempferol	130.16 ± 4.78	-	-
Longan	Epicatechin	25.68 ± 0.99	Epicatechin	12.92 ± 0.54
	Kaempferol	154.12 ± 10.06	Gallic acid	4.36 ± 0.30
Mango	Gallic acid	5.36 ± 0.40	Gallic acid	3.84 ± 0.20
	Chlorogenic acid	49.20 ± 2.45	Kaempferol	155.48 ± 4.34
	Cyanidin 3-glucoside	6.12 ± 0.40	-	-
	Homogentisic acid	23.40 ± 0.95	-	-
	Kaempferol	155.48 ± 6.96	-	-
Starfruit	Catechin	76.36 ± 5.42	Gallic acid	4.36 ± 0.30
	Epicatechin	172.08 ± 6.94	Galangin	104.76 ± 5.23
	Forulic acid	52.92 ± 2.85	-	-
	Gallic acid	17.04 ± 1.14	-	-
	Kaempferol	33.68 ± 2.33	-	-
Sweetsop	Catechin	143.72 ± 4.22	Catechin	28.80 ±1.34
	Cyanidin 3-glucoside	21.00 ± 0.88	Kaempferol	149.76 ± 5.63
	Epicatechin	164.60 ± 4.30	-	-
	Protocatechuic acid	26.76 ± 1.10	-	-
Ziziphus jujuba	Gallic acid	10.00 ± 0.50	Catechin	35.16 ± 2.22
	Kaempferol	160.92 ± 10.55	Kaempferol	125.80 ± 6.40

**Table 7:** Comparison of the antioxidant activity levels and phenolic contents of fat- and water-soluble fractions.

Sample	Item	Fraction	No.	Mean ± SD	Median (25th, 75th)	*Z*	*p*
Peel	ABTS	fat-soluble	47	15.26 ± 13.70	10.89 (4.76, 20.06)	−5.108	<0.001
		water-soluble	47	7.61 ± 10.93	3.15 (0.8, 6.53)		
	FRAP	fat-soluble	47	16.07 ± 22.36	7.41 (3.59, 18.40)	−3.132	0.002
		water-soluble	47	11.58 ± 15.49	6.66 (3.11, 9.60)		
	TPC	fat-soluble	47	3.66 ± 2.54	3.11 (2.72, 4.15)	−5.334	<0.001
		water-soluble	47	1.72 ± 2.02	1.12 (0.65, 1.91)		
Seed	ABTS	fat-soluble	33	13.95 ± 12.43	9.77 (6.16, 15.39)	−4.806	<0.001
		water-soluble	33	5.52 ± 9.89	2.01 (0.10, 6.04)		
	FRAP	fat-soluble	33	12.20 ±17.17	7.31 (3.48, 13.77)	−3.478	0.001
		water-soluble	33	7.98 ± 17.07	3.90 (1.77, 6.57)		
	TPC	fat-soluble	33	3.47 ± 2.29	2.87(2.35, 3.97)	−4.941	<0.001
		water-soluble	33	1.29 ± 1.95	0.83 (0.45, 1.07)		

A calculated P value was considered to be statistically significant difference at level of 5%.
